# Non-COVID-19 general practice and our response to the pandemic

**DOI:** 10.3399/bjgpopen20X101095

**Published:** 2020-05-20

**Authors:** Dipesh P Gopal

**Affiliations:** 1 Honorary Research Fellow in Primary Care, and General Practitioner, Institute of Population Health Sciences, Barts and The London School of Medicine and Dentistry Blizard Institute, London, UK

**Keywords:** Family medicine, community care, infectious illness, primary health care

Clinicians in family medicine look after the health of patients with different biopsychosocial problems. During the pandemic, I worry about the care of patients with multimorbidity and, specifically, the widespread cancellation of routine secondary care for many patients. Notably, some patients with cancer have their surgery and non-surgical therapy cancelled indefinitely, which potentially risks disease progression.^1^ While treatment is meant to be assessed on a case-by-case basis, we know that the majority of healthcare resources are being diverted to fight the pandemic.^2^ Routine outpatient clinics are cancelled and only urgent diagnostic clinics, such as those to detect suspected cancer, remain open. Myopic vision may very well miss the bigger picture of non-COVID-19 health care. Similar concerns have been voiced for tuberculosis services, where diagnostic and treatment services have been disrupted,^3^ but could apply to communicable and non-communicable disease alike. While this may pose a challenge, there is an opportunity to focus on what matters to patients in primary care rather than low-impact tick-box bureaucracy and payment for performance (P4P) targets.^4^

While our current focus is undoubtedly on the pandemic, we must plan for and imminently anticipate a further resource-depleted healthcare system as well as the impact of lack of routine non-communicable disease care, which is the mainstay of family medicine. In England, March marked the end of the ‘financial year’ for P4P or Quality and Outcomes Framework (QoF), so it is likely many chronic disease care checks were complete.

As a global community, we will undoubtedly be struck by the consequences of socioeconomic turmoil,^5,6^ such as worsening mental health conditions, homelessness, and poverty. Krist and colleagues^5^ mention the need for rehabilitation for those who have had treatment for COVID-19, and anticipate worsening of existing long-term conditions. Those families who were unable to visit their unwell relatives in their final days with COVID-19^7^ may suffer post-traumatic stress disorder and prolonged grief or bereavement disorders. Finally, many healthcare workers are likely to suffer psychological consequences such as grief from the death of colleagues, stress, fatigue, and burnout from demanding work conditions.

This is a lot to take in; there are few guidelines to help prepare primary care for the consequences of a pandemic that is quite literally a ‘once in a generation’ catastrophe. We must support one another and create both physical and virtual space as well as time for healthcare workers to discuss their experiences. Given the inverse-care law, it would be sensible to prioritise chronic disease reviews for those that live in the most deprived locations of your practice catchment areas.^8^ Parts of chronic disease reviews may be done remotely to decrease treatment burden. Rehabilitation and mental health problems will require support from other parts of healthcare systems, but this should not stop us pursuing innovative ways of support such as online interventions. We must be aware of the rise of domestic violence during the imposed restrictions of staying home. During the ‘lockdown’, French pharmacies use codewords to provide victims of domestic abuse a place of safety and perhaps we could similarly use different members of our communities to provide this in future.^9^ The Health Select Committee^10^ suggested that in some areas the number of referrals for suspected cancer had decreased by up to 75%, which potentially means later presentations of cancer and worse prognoses on lifting of pandemic restrictions. The biggest challenge of going into post-pandemic Britain will undoubtedly be socioeconomic. Primary care can provide a solution to some medical and psychological problems, but socioeconomic problems will require socioeconomic solutions. In February 2020, the Marmot report^11^ showed that life expectancy had slowed down and that health inequalities between the richest and poorest members of British society had widened over the last decade. This coincided with cuts to public funding. This was the situation going into the pandemic, and the divide between the richest and poorest is likely to widen further post-pandemic. I propose two solutions.

The first is bottom-up, through our communities, and we should hold onto the momentum of 750 000-strong British volunteer force raised during the pandemic which may represent early signs of renewed social cohesion. Before the pandemic, the establishment of the National Academy of Social Prescribing (NASP) heralded a new age of linking up members of society to improve their health and wellbeing by giving members of the public access to meaningful activities. Social prescribing could ensure that the marginalised members of our society are not forgotten. However, there is limited evidence from a systematic review^12^ of the benefit of social prescribing due to differences in study methodology. The other way is top-down and will require political backing to adequately fund local authorities to help support housing for those who are the poorest in our society. The most deprived populations have the shortest life expectancy and longest time spent in disease compared to least deprived populations. Asking for government funding will prove difficult as the global economy shudders back into life after the pandemic. I hope that governments worldwide do not forget the importance of well-funded and well-resourced state-backed services, such as healthcare and community infrastructure, both inside and outside a pandemic.
